# Artificial Diets for Mosquitoes

**DOI:** 10.3390/ijerph13121267

**Published:** 2016-12-21

**Authors:** Kristina K. Gonzales, Immo A. Hansen

**Affiliations:** 1 Department of Biology, New Mexico State University, Las Cruces, NM 88003, USA; 2 Institute of Applied Biosciences, New Mexico State University, Las Cruces, NM 88003, USA

**Keywords:** artificial blood meal, *Aedes aegypti*, mosquito, diet

## Abstract

Mosquito-borne diseases are responsible for more than a million human deaths every year. Modern mosquito control strategies such as sterile insect technique (SIT), release of insects carrying a dominant lethal (RIDL), population replacement strategies (PR), and *Wolbachia*-based strategies require the rearing of large numbers of mosquitoes in culture for continuous release over an extended period of time. Anautogenous mosquitoes require essential nutrients for egg production, which they obtain through the acquisition and digestion of a protein-rich blood meal. Therefore, mosquito mass production in laboratories and other facilities relies on vertebrate blood from live animal hosts. However, vertebrate blood is expensive to acquire and hard to store for longer times especially under field conditions. This review discusses older and recent studies that were aimed at the development of artificial diets for mosquitoes in order to replace vertebrate blood.

## 1. Introduction

Disease-vectoring mosquito species can acquire and transmit pathogens that cause human diseases such as malaria, Dengue fever, yellow fever, chikungunya, Zika, West Nile, Japanese encephalitis, and lymphatic filariasis [1,2]. The highest incidence of these mosquito-borne diseases is located in the tropical and subtropical parts of the world with over 500 million cases reported yearly [1]. Recent outbreaks of the mosquito-transmitted Zika virus in the Americas have caused a state of emergency due to an association between Zika infection and microcephaly [3]. Although effective vaccines for yellow fever and Japanese encephalitis have been developed [4], there are currently no approved vaccines for the most important mosquito-borne diseases, malaria and dengue fever, and medical interventions focus solely on treating the symptoms. Potential vaccines for malaria and dengue are currently undergoing clinical trials and the development of other vaccines is underway, however successful outcomes of some of these projects is circumspect [1,5]. Mosquito population control using public health insecticides has historically been the most effective way to control mosquito-borne diseases when vaccines are not available. Increasing insecticide resistance in vector populations has seriously decreased the efficacy of this approach [6,7,8].

### 1.1. Eco-Friendly Mosquito Control Strategies Require Mass Production of Mosquitoes

Novel, innovative mosquito control methods such as sterile insect technique (SIT), release of insects carrying a dominant lethal (RIDL), population replacement strategies (PR), and *Wolbachia* endosymbiont-driven techniques offer “green”, eco-friendly alternatives to insecticides [9,10,11,12,13,14,15,16,17,18,19,20,21,22]. The implementation of these biocontrol methods requires the use and release of sterilized or genetically modified mosquitoes that are generated and released in mass amounts in order to interact with and/or replace wild populations. Artificial diets for adult female mosquitoes could play an important role in supporting these novel approaches to control mosquito populations and mosquito-borne diseases by reducing costs and alleviating the problems associated with the use of vertebrate blood (see below).

### 1.2. Vertebrate Blood-Based Mosquito Culture

**Figure 1:**
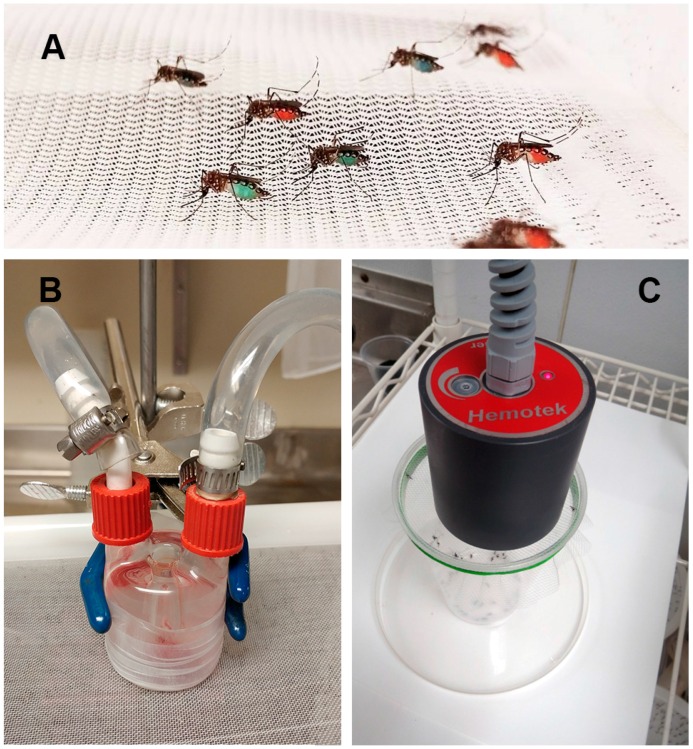
Artificial feeding systems for mosquitoes. (**A**) *Aedes aegypti* mosquitos engorged on an artificial blood meal replacement diet. Food colors were added to the different SkitoSnacks; (**B**) Glass membrane feeder for mosquitoes. Warm water is used in this device to keep the meal at body temperature. Mosquitoes suck the meal through a Parafilm^®^ membrane (Sigma Aldrich, St. Louis, MO, USA); (**C**) Hemotek feeding system (Hemotek Ltd., Great Harwood, UK).

Most disease-transmitting mosquito species are anautogenous, meaning that the females require a vertebrate blood meal for egg development. Current mosquito laboratory rearing practices require the use of sedated or restrained live animals as a blood source, usually mice, rats, or chickens. This imposes complications to the laboratory setting such as: compliance with the Institutional Animal Care and Use Committee policies, personnel training, animal housing and care, facility maintenance, equipment and reagent expenses. Ethics concerning animal welfare are also a major constraint [23,24,25]. An alternative that is also very common is the use of purchased, isolated blood that has been treated to prevent clotting. Whole vertebrate blood can be purchased from various sources and fed to mosquitoes via different contraptions. Water-jacketed artificial membrane feeders [26] or similar devices, for example the Hemotek feeding system (Hemotek Limited, Great Harwood, UK), are common (see Figure 1). Several protocols have been published describing mosquito culture and methods to feed blood to them [27,28,29].

Both methods, live animals and isolated blood, have disadvantages. In many countries significant administrative efforts are necessary in order to get appropriate permissions to use live animals and the sedatives used might have an influence on mosquito biology. Donated human blood is even more problematic to use in mosquito culture since it carries the risk of blood-borne pathogens and underlies more stringent regulations than animal blood [30,31]. Obtaining and storing vertebrate blood is also costly and difficult. The shelf life for blood is approximately two weeks, blood is not always of consistent quality, and it requires constant cooling. Therefore, the acquisition of “fresh” vertebrate blood constitutes a serious bottleneck when considering the large-scale implementation of the above mentioned mosquito control techniques in the field.

### 1.3. Requirements for Artificial Mosquito Diets

We hypothesize that the formulation and use of artificial diets for mosquito rearing can help reduce costs, effort, and eliminate live animal use for mosquito culture in the long term. In order to replace vertebrate blood, an artificial blood meal should meet the following standards [32]:

(1) Females must readily ingest the meal in sufficient amounts.
(2) It must support vitellogenesis.
(3) It must support large egg batches.
(4) The competitiveness of offspring should be comparable to wild mosquitoes.
(5) Mosquito behavior and immunity should not be affected.
(6) It must not interfere with *Wolbachia* symbionts (only important for *Wolbachia* endosymbiont-driven techniques).

The first mosquito artificial blood meal replacements were introduced, formulated, and tested in the early 1900s [33]. A plethora of studies have been published since then. Below, we will explore the history and recent developments of artificial mosquito diets and their implications.

### 1.4. Nutritional Regulation of Mosquito Egg Development

As mentioned above, anautogenous female mosquitoes need vertebrate blood in order to provide their first batch, and subsequent batches, of eggs with nutrients. Autogenous mosquitoes in contrast can mobilize nutrients accumulated during the larval phase and produce a first batch of eggs without taking blood [34,35]. Culture of such species or strains can be accomplished without the use of blood or artificial diets. An extensive discussion of these two reproductive strategies can be found in the review of Attardo and coworkers [36]. The major human disease-vectoring mosquito species are anautogenous.

Vertebrate blood is rich in proteins that are digested, in the mosquito midgut, by trypsin-proteases into their constituent amino acids [37,38,39,40]. Free amino acids are transported out of the midgut and taken up by the fat body and other tissues via specialized amino acid transporters, where specific signaling pathways in these tissues are subsequently activated [41,42,43,44,45]. A large percentage of the blood-meal derived amino acids is metabolized and used for energy production while the rest is used for the massive synthesis of yolk proteins that starts shortly after a blood meal [46,47]. Once yolk proteins are synthesized by the mosquito fat body, they are released into the hemolymph, the insect open circulatory system, and then deposited in oocytes via receptor-mediated endocytosis [46,48]. This entire process is termed vitellogenesis. For a summary on the regulation of vitellogenesis, please see our 2014 review on this topic [49].

Attardo and coworkers found that the fat body of *Aedes aegypti* requires a balanced mixture of amino acids in order to initiate vitellogenesis. Withdrawal of a single essential amino acid from the mix resulted in a strong reduction in yolk protein gene expression [50]. This data implies that an artificial blood meal replacement diet for mosquitoes has to contain sufficient and balanced amounts of essential amino acids in order to successfully support egg development. Diets that are based on a single protein source (such as bovine serum albumin (BSA)) might have to be supplemented with some essential amino acids that this protein lacks. 

### 1.5. Mosquito Host Preferences

Different mosquito species prefer certain vertebrate blood sources over others [51]. Host preference is a complex phenomenon which involves intrinsic factors, such as the sensory apparatus that mosquito females use for host seeking, as well as behavior components and extrinsic factors like the density of the host species [52]. Host preference has implications for the development of artificial blood meal replacement diets, because the nutritional composition of blood from different vertebrate animal sources can be quite different. As a consequence, egg numbers produced after taking a full blood meal can also vary significantly depending on the host species. Mosquitoes do not by default prefer the host species whose blood produces the optimal reproductive output.

Suleman and Shirin (1981) have shown that *Culex quinquefasciatus* feed more readily on warm-blooded vertebrates (mouse, rabbit, and pigeon) than cold-blooded vertebrates (lizard and toad) [51]. Interestingly, the mosquitoes that fed on the lizard blood produced the most eggs per raft (140.8 ± 6.73) when compared to the other vertebrate blood sources. However, there were no significant detectable differences in hatch rates for all blood sources. In another study, Bennett (1970) found that *Ae. aegypti* produced more eggs, on average, from avian blood than from mammalian blood when fed through an artificial membrane feeder [53]. His findings suggest that avian blood is superior in nutritional value when compared to other mammalian vertebrate blood sources. Richards et al. (2012) found that the blood source and blood anticoagulant used for colony maintenance has an effect on the fecundity and fertility rates of *Culex quinquefasciatus* [54]. Feeding on a live chicken produced the highest mean number of eggs per raft (122 ± 5.9), and mean number of larvae hatched per raft (101 ± 6.0). The fecundity and fertility rates decreased in all feeding trials during the second gonotrophic cycle, which may indicate that female nutritional reserves are depleted with age and each gonotrophic cycle. Interestingly, defibrinated bovine blood and citrated bovine blood feeding trials were not significantly different from each other in terms of fecundity and fertility during the first gonotrophic cycle. However, the mean number of eggs per raft and mean number of larvae per raft was less than half of that of the live chicken, suggesting that a live, avian animal source is best. Phasomkusolsil et al. (2013) tested the influence of the blood source on the life history traits of *Anopheles dirus*, *An. cracens*, *An. minimus*, *An. sawadwongporni*, and *Ae. Aegypti* [55]. All mosquito species were fed on each of the following blood sources: anesthetized hamster, defibrinated sheep blood, defibrinated guinea pig blood, and human blood. All vector species had a preference towards a particular host. *An. dirus* engorged the most on guinea pig and human blood, *An. cracens* and *An. minimus* preferred the blood of a live hamster, guinea pig, and sheep blood. *An. sawadwongporni* fed on sheep blood most frequently and *Ae. aegypti* preferred the live hamster and sheep blood. Interestingly, only *An. dirus* engorged on human blood at a higher percentage (97.0 ± 1.0) than the other species (48.7 ± 7.0 to 71.7 ± 9.3 percent range).

Several of the above mentioned studies showed that mosquitoes fed on avian blood produced more eggs that those fed on mammalian hosts. One possible reason for this observation is that avian red blood cells are nucleated and therefore avian blood contains much higher concentrations of DNA compared to mammalian blood. However, we are not aware of any experimental evidence supporting this hypothesis. 

## 2. Requirements for Mosquito Vitellogenesis

### 2.1. Proteins in Artificial Mosquito Diets

It has been established that protein in mosquito meals is an essential requirement for egg development [56]. Early studies have identified different diets and protein sources that can support this process (see Table 1).

Protein is not only a basic requirement for oogenesis, but also the concentration of protein in the meal is important. Huff (1929) raised *Culex pipiens* larvae on diluted milk and the resulting adults were fed on various meals [33]. He found that ovulation and egg viability was possible when adult females were fed on hemoglobin, egg yolk, ox blood serum, blood peptone, cell broth, and various undiluted vegetable and fruit juices. Lea et al. (1955) found that *Aedes aegypti* and *Ae. quadrimaculatus* were able to produce viable eggs when they fed on a solution of honey and skimmed milk or honey and blood [57]. In the same study, *Ae. quadrimaculatus* produced viable eggs when fed another solution composed of proteose-peptone, liver concentrate, and casein protein. Kogan (1990) fed *Ae. aegypti* a meal made of three porcine proteins (γ-globulins, hemoglobin, and albumin) dissolved in a sodium chloride/sodium bicarbonate solution supplemented with adenosine triphosphate [56]. He found that increasing the protein concentration of his substitute meal from 60 mg/mL to 125 mg/mL substantially increased egg yield. He also determined that females produced the same egg yield when fed on either the 125 mg/mL total protein substitute meal or whole blood. Cosgrove and Wood (1996) sought to improve Kogan’s formula for *Ae. aegypti*, *Anopheles arabiensis*, and *An. stephensi* by testing various meal formulations that utilized bovine, porcine, or both animal proteins dissolved in water or Ringer’s solution [59]. These protein formulations contained various concentrations of globulin, hemoglobin, and albumin. Griffith and Turner (1996) was able to rear 15 consecutive generations of *Culex quinquefasciatus* on an artificial diet composed of sodium chloride, sodium bicarbonate, soy milk formula, ovalbumin, gamma-globulins, and adenosine triphosphate (ATP). They conclude that the offspring produced from live guinea pigs and their artificial diet were comparable in their life traits (weight, sex ratio, lifespan, and fertility) [60]. Pitts (2014) formulated a blood-free meal for *Ae. albopictus* that consisted of 100 mg/mL BSA or 200 mg/mL BSA in a phosphate buffered saline solution (PBS) [61]. He recorded an average of 57 eggs produced in females fed the 100 mg/mL solution compared to 92.2 eggs produced in the females fed the 200 mg/mL meal. He also performed a single trial experiment where he fed *Ae. albopictus* a 400 mg/mL BSA meal and found that there was no increase in egg production, thus, he concluded that the 200 mg/mL BSA meal contained a sufficient amount of protein to support vitellogenesis. Pitts was able to maintain *Ae. albopictus* colonies for six consecutive generations on the artificial diet. Gonzales et al. (2015) tested Pitts’ 200 mg/mL BSA/PBS meal and compared it to whole bovine blood, washed bovine red blood cells, serum, and commercially available bovine hemoglobin, to determine whether vitellogenesis and offspring viability can be supported in *Ae. aegypti* [32]. She found that the BSA/PBS meal was comparable to whole bovine blood in terms of engorgement rates, and the number of eggs laid and retained in the ovaries. However, hatch rates for this meal were low indicating a lack of an important nutrient for viability. Talyuli et al. (2015) formulated a chemically-defined diet containing bovine proteins (hemoglobin, albumin, and γ-globulins) in Tyrode’s buffer supplemented with cholesterol to determine the role of dietary cholesterol [62].

### 2.2. Hemoglobin in Artificial Mosquito Diets

Hemoglobin makes up more than 90% of the dry mass of vertebrate blood [63]. It is therefore not astonishing that this iron-containing protein has been included in many experimental artificial mosquito diet recipes. Hemoglobin was used as a visual marker in Kogan’s meal (1990) [56]. In a follow up study, Cosgrove and Wood (1996) tested a meal consisting of bovine albumin (100 mg/mL) and globulin (30 mg/mL) in Ringer’s solution with or without 8 mg/mL of hemoglobin and found that hemoglobin supplementation was associated with increased egg production and egg hatch rates in *Ae. aegypti* [59]. Conversely, Gonzales and coworkers (2015) found that a meal containing only washed bovine red blood cells, predominately hemoglobin proteins, or bovine hemoglobin (200 mg/mL of dried bovine red blood cells dissolved in phosphate buffered saline) failed to support egg production in *Ae. aegypti* [32]. Hemoglobin at higher concentrations also acted as a phagosuppressor, significantly reducing engorgement rates in a concentration-dependent manner. However, in follow up studies, Gonzales found that supplementation of hemoglobin, at a lower concentration, significantly increased egg viability in *Ae. aegypti* [64]. These findings suggest that hemoglobin is important for egg viability but that its concentration in artificial blood meal replacements for mosquitoes has to been carefully adjusted. 

### 2.3. Micronutrients in Artificial Mosquito Diets

Iron—It has become apparent that iron is an especially important micronutrient for mosquitoes. When adult female mosquitoes take a blood meal they ingest high concentrations of iron bound to heme in hemoglobin. Zhou and coworkers (2007) studied the fate of iron acquired via blood feeding in *Ae. Aegypti* [65]. They added radiolabeled iron to pig blood and fed it to *Ae. aegypti* to determine where blood-meal iron accumulates after the first gonotrophic cycle. Interestingly, the majority of blood meal heme iron (87%) is excreted immediately after blood feeding, but the portion of iron that is absorbed (13%) is distributed in the fat body, head, ovaries, and eggs of the mosquito. Over half of the absorbed iron is translocated to the eggs. They also found that radiolabeled iron bound to ferritin was retained by 15% and most of it was found in the eggs (~77%). Gonzales and coworkers (2014) found that the addition of iron(III)chloride to a BSA-based artificial diet significantly increased egg viability and hatch rates from 15.2 ± 5.2 to 33.1 ± 12.3 [32].

Recently, our laboratory has performed a series of experiments where we fed *Aedes aegypti* a BSA meal supplemented with 30 µM of various iron sources—iron (II) bisglycinate, iron (III) chloride, iron (II) citrate, iron (III) citrate, iron (II) fumarate, iron (II) gluconate, and hemoglobin. Females produced the most eggs, on average, when fed on iron (III) citrate. When no iron was present in the meal, the average viability percentage was very low. However, the addition of iron salts or hemoglobin increased the average viability percentage and the addition of iron (II) bisglycinate doubled it [64]. The results of these studies suggest that iron supplementation is a critical factor, essential for the success of artificial blood meal replacements. 

Sugars—Adult mosquitoes of both sexes ingest sugary solutions when available. For example, *Aedes aegypti* are reported to acquire sugar from plant sources for energy before mating and throughout their adult life [66,67,68]. Sugar solution is stored in the crop, not the midgut, and is utilized for energy production [69]. The predominate sugars found in floral nectars are fructose, glucose, and sucrose [66]. Ignell et al. (2010) compared the acceptance of glucose, fructose, sucrose, and trehalose of *Ae. aegypti* of both sexes and found that acceptance was directly related to concentration [67]. It is standard practice in the laboratory culture of various mosquito species to provide them with sucrose solutions at concentrations between 10% and 20% (*w*/*v*). To date, the effects of sugar supplementation in artificial blood-meal replacements on mosquito life span, egg numbers, or egg hatch rates have not been explored. However, the strong phagostimulatory effects on mosquitoes that some sugars exhibit (see below) provide a strong rationale to add them to artificial blood meal replacement diets.

Cholesterol—Talyuli et al. (2015) generated an artificial blood meal replacement based on the protocols of Kogan (1990) and Cosgrove and Wood (1996) for *Ae. aegypti.* They added cholesterol-packed micelles to determine their effects on female egg development in adults that were raised as nutritionally-deficient, small mosquitoes, and nutritionally-adequate, large mosquitoes [62]. They found that feeding their artificial blood meal replacement resulted in an increase in small mosquito egg deposition by 80% while the large mosquitoes produced egg numbers comparable to rabbit blood controls. These results suggest that the addition of cholesterol to artificial blood meal replacements can have a strong positive effect on the outcomes of feeding such diets.

A study by Caragata and coworkers found that *Wolbachia* infection reduces total cholesterol levels in mosquitoes by up to 25% [70]. Cholesterol supplementation of a blood meal did not improve egg numbers or viability. However, dietary cholesterol has been shown to have a modulatory effect on pathogen blocking by *Wolbachia* in the fruit fly *Drosophila melanogaster* [71]. *Wolbachia*-infected flies reared on high-cholesterol diets showed reduced pathogen blocking after being challenged with Drosophila C virus. Therefore, the effects of varying levels of cholesterol in artificial blood meal replacement diets on the offspring mosquitos susceptibility to arbovirus infections has to be thoroughly explored before such diets can be used to rear mosquitoes for *Wolbachia*-based interventions. 

Amino acids—Several studies have shown that certain amino acids are important for vitellogenesis. Uchidi and coworkers showed that infusion of a 17 amino acid mixture in the hemocoel of different anautogenous mosquito species resulted in the maturation of egg follicles [72]. As mentioned above, Attardo and coworkers (2006) discovered that ten amino acids are essential for vitellogenic gene transcription [50]. Those amino acids were: leucine, tryptophan, methionine, valine, histidine, lysine, phenylalanine, arginine, asparagine, and threonine. The amino acid isoleucine has also been linked in several studies to higher egg production in mosquitoes. Dimond et al. (1955) developed a meal containing 18 amino acids (including isoleucine) in 10% honey water, at the concentrations present in blood [58]. They found that this solution could produce 2600 eggs from a batch of 500 *Aedes aegypti* females, a rather low number. A similar result was achieved by the same group in the following year with a twelve amino acid mixture that also included isoleucine [73]. In his paper on an artificial diet for *Aedes aegypti*, Kogan (1990) mentions that the whole bovine blood that he used as his control had to be supplemented with 2 mg/mL of isoleucine because bovine blood alone is low in isoleucine [56]. Cosgrove and Wood (1996) supplemented Kogan’s original meal, containing porcine proteins, with 2.5 mg/mL isoleucine. Isoleucine supplementation increased egg numbers and pupal yield [59]. They also concocted and tested another meal made with bovine albumin, hemoglobin, and γ-globulin proteins dissolved in Ringer’s solution supplemented with 2.5 mg/mL of isoleucine and found the opposite effect—egg numbers, hatch, and pupal yield decreased upon addition of isoleucine. Harrington (2001) found that human blood containing low and high levels of isoleucine were not statistically significantly different from each other in terms of the number of eggs produced per mosquito [74]. The studies discussed above indicate that specific essential amino acids are critical for the success of artificial mosquito diets. Therefore supplementation of some amino acids might be necessary especially if the major protein source in a particular artificial diet lacks or only contains insufficient concentrations of it.

### 2.4. Phagostimulants in Artificial Mosquito Diets

Phagostimulants are compounds present in a meal that are detected by insect chemoreceptors. Their presence encourages insects to imbibe their meals to full engorgement. Female mosquitoes detect blood with the labrum, which is a sense organ located on top of the mosquito food canal [66].

Sodium Chloride—Hosoi (1959) found that sodium chloride at 150 mM acted as a phagostimulant for *Culex pipiens* mosquitoes [75]. Engorgement rates of about 70% were observed while there was no feeding on distilled water [75,76]. A study on anopheline mosquitoes found that *An. stephensi*, *An. freeborni,* and *An. dirus* imbibed artificial meals containing sodium chloride and sodium bicarbonate, indicating that these chemicals are phagostimulatory [77].

Adenosine Triphosphate—Today, the most effective phagostimulant known for mosquitoes and a variety of other blood-sucking arthropods is adenosine triphosphate (ATP) [77,78,79,80,81]. Galun (1967) tested various artificial meals to determine what adenine nucleotides, present in blood, encouraged engorgement and determined that the feeding percentage of each meal increased with the number of attached phosphates in the following order: adenosine monophosphate, adenosine diphosphate, adenosine triphosphate (ATP), and adenosine tetraphosphate at 1 mM [76]. In these experiments, the artificial solutions were warmed to at least 37 °C. Interestingly, Galun (1985) also found that the concentration of ATP did not have any relationship with engorgement response and that four *Anopheles* species do not require ATP as a phagostimulant [77]. Conversely, *Ae. aegypti* feeding response was directly related to ATP concentration, suggesting that this species requires ATP for engorgement. Griffith and Turner (1996) also found that ATP at 1 mM was necessary to induce engorgement on their simple sodium chloride and sodium bicarbonate solution by *Aedes aegypti*, *Culex quinquefasciatus*, and *Anopheles arabiensis* [60].

Pitts (2014) tested serial dilutions of ATP in his artificial blood meal at 0 mM, 0.001 mM, 0.01 mM, 0.1 mM, and 1 mM concentrations. He found that ATP at 1 mM produced the best engorgement response (69%) comparable to whole human blood (59%) [61].

Sugars—Hosoi (1959) performed feeding to determine what components of blood induce mosquito gorging. He found that sweetening his saline solution with 2%–5% of glucose increased full engorgement of *Culex pipiens* [75]. Ignell et al. (2010) performed a two choice assay to determine diet selection in *Ae. aegypti*. They tested two concentrations (10 µM and 10 mM) of the 20 amino acids combined with two concentrations (10 µM and saturation) of sucrose and found that *Ae. aegypti* preferred certain amino acid(s)/sucrose combinations in the diet over sucrose offered alone [67]. Recently, our lab has tested the phagostimulatory effects of naturally occurring mono- and disaccharides and compared them to whole bovine blood and 3 mM ATP [64]. We have tested engorgement rates of 50 mM, 100 mM, and 1 M concentrations of arabinose, fructose, galactose, glucose, sucrose, and trehalose. We found that a BSA meal alone, without sugar, had a 21% engorgement rate, ATP had the highest engorgement rates from 85%–90%, and 50 mM and 100 mM glucose had the second highest engorgement rate at 41% and 25%, respectively. Engorgement rates for the remaining sugars at 50 mM and 100 mM ranged from 15%–30% and 14%–20%, respectively.

These results show that phagostimulants are an essential part of artificial blood meal replacement diets. ATP is currently the standard producing the highest engorgement rates (in *Aedes* mosquitoes). However, ATP cannot be stored at room temperature and is relatively unstable in aqueous solutions. Finding a replacement compound for ATP is therefore desirable.

### 2.5. The Price of Artificial Blood Meals

Maintaining a culture of an anautogenous mosquito species at this time requires a constant supply of vertebrate blood, which can be quite expensive. Therefore, the price of artificial blood meals is of interest. It is noteworthy that artificial blood meal replacements may in fact reduce operational costs for mosquito culture by removing the need for animal husbandry, etc., even if the actual purchase costs for their ingredients is more than that of blood.

The artificial blood meals used by Pitts (2014) and Gonzales et al. (2015) used BSA, fraction V (Research International Products) at 200 mg/mL as the main protein source [32,61]. BSA is the price-driving ingredient in these diets because the other ingredients are added at relatively small concentrations. The cost per milliliter will depend on the amount of BSA purchased and varies from $0.17–$0.34. Purchasing whole blood can be costly depending on the animal source and method of blood treatment. Current costs per milliliter for defibrinated blood (Hemostat Laboratories) from selected species are: bovine, $0.14–$0.60; sheep, $0.05–$0.38; and rabbit, $0.39–$0.73. Therefore, the unit price of whole blood is comparable to the cost of a BSA-based artificial diet. However, whole blood has a shelf life of two to four weeks, depending on the anticoagulant used, and it must be purchased frequently which increases the overall cost. Also, the animal source of blood or anticoagulant might have an impact on mosquito fecundity and viability [51,54,55]. This can lead to inconsistent results that may impact colony maintenance and research productivity. Therefore, in addition to being cost-effective, an artificial blood meal that is chemically defined will provide reliable and consistent nutrition to mosquitoes at the adult stage. The implementation of an artificial blood meal would be in compliance with the “3 R’s: replacement, reduction, and refinement” of animal welfare regulations, which require institutions to consider and utilize other non-animal protocols, limit the number of animals used, and lessen unnecessary distress in laboratory animals [24].

## 3. Conclusions

With the current rise in interest in eco-friendly mosquito control techniques, the interest in artificial blood meal replacement diets for mosquitoes has increased. The experimental studies we discussed above suggest that such diets could become effective replacements for vertebrate blood in mosquito mass culture. However, these diets must be carefully tested and possibly adapted to each mosquito species and their effects on mosquito biology should be studied before they can be broadly used.

## Figures and Tables

**Table 1:** A selection of publications on artificial blood meal replacement diets for mosquitoes.

Citation	Mosquito Species	Diet Components	Success Rate
1955. Lea et al. [57]	*Aedes aegypti*, *Ae. quadrimaculatus*	Skimmed milk with 10% honey.	The authors state that skimmed milk can be a substitute for blood to produce eggs. *An. quadrimaculatus* was also fed proteose-peptone, liver concentrate, and casein hydrolysate.
1955. Dimond et al. [58]	*Aedes aegypti*	Mixture of 18 amino acids in 10% honey water.	A low egg number of 5.2 eggs/female is reported
1990. Kogan [56]	*Aedes aegypti*	Porcine albumin (102 mg/mL), γ-globulin (15 mg/mL), hemoglobin (8 mg/mL), ATP (1 mM), NaCl (5–10 mM), and NaHCO_3_ (120 mM) in water.	101 ± 6.8 eggs/female
1996. Cosgrove and Wood [59]	*Aedes aegypti*	Porcine albumin (100 mg/mL), γ-globulin (15 mg/mL), hemoglobin (8 mg/mL), isoleucine (2.5 mg/mL), ATP (1 mM), NaCl (5–10 mM), and NaHCO_3_ (120 mM) in water.	55.32 ± 4.1 eggs/female. 61.9% hatch rate
1996. Cosgrove and Wood [59]	*Aedes aegypti*	Bovine serum albumin (100 mg/mL), γ-globulin (30 mg/mL), hemoglobin (8 mg/mL), and ATP (1 mM) in Ringer‘s solution.	64.56 ± 5.1 eggs/female. 54.1% hatch rate. A colony of *Ae. aegypti* was raised on this diet for more than 25 generations
1996. Griffith and Turner [60]	*Culex quinquefasciatus*	Soy milk formula (33 mg/mL), γ-globulin (11.5 mg/mL), ovalbumin (76.5 mg/mL), ATP (1 mM), NaCl (150 mM), and NaHCO_3_ (100 mM) in water.	116 eggs/female. Colonies were maintained for 6 and 15 generations
2014. Pitts [61]	*Aedes albopictus*	Bovine serum albumin (200 mg/mL) and ATP (1 mM) in phosphate buffered saline.	92.2 ± 4.4 eggs/female. 71.8% ± 6.1% hatch rate. Colonies were maintained for 6 generations
2015. Gonzales et al. [32]	*Aedes aegypti*	Bovine serum albumin (200 mg/mL), ATP (1 mM), and iron (III) chloride (0.5 mg/mL) in *Aedes* physiological saline.	66 ± 10.8 eggs/female. 33.06% ± 12.34% hatch rate
